# Formation of Complexes Between O Proteins and Replication Origin Regions of Shiga Toxin-Converting Bacteriophages

**DOI:** 10.3389/fmolb.2020.00207

**Published:** 2020-08-19

**Authors:** Katarzyna Kozłowska, Monika Glinkowska, Lidia Boss, Lidia Gaffke, Jakub Deptuła, Grzegorz Węgrzyn

**Affiliations:** ^1^Department of Molecular Biology, Faculty of Biology, University of Gdańsk, Gdańsk, Poland; ^2^Department of Bacterial Molecular Genetics, Faculty of Biology, University of Gdańsk, Gdańsk, Poland

**Keywords:** Shiga toxin-converting bacteriophages, DNA replication, replication initiator protein, replication complex, protein-DNA interactions

## Abstract

Shiga toxin-converting bacteriophages (or Stx phages) are responsible for virulence of enterohemorrhagic *Escherichia coli* strains. Although they belong to the group of lambdoid phages, which have served as models in studies on DNA replication mechanisms, details of regulation of replication of Stx phage genomes are poorly understood. Despite high similarity of their replication regions to that of phage lambda, considerable differences occur between them. Here, we present a comparison of origins of replication and O proteins of lambda and selected Stx phages (phages P27 and 933W). Stx initiator proteins, similarly to the lambda O protein, exist in the form of dimers. Only 4 iteron sequences are strongly bound *in vitro* by the O proteins, despite the presence of 6 such fragments in the Stx *ori*, while the function of the other two iterons is still crucial for transformation of *E. coli* wild-type strain by the P27-derived lambdoid plasmid. As these sequences are found in the gene coding for Stx O proteins, the sequences of these proteins themselves are also extended compared to lambda phage. Therefore, proteins O of Stx phages P27 and 933W have 13 additional amino acids. They can act as a space barrier, thus affecting the lesser packing of the O-some Stx complex compared to the structure found in lambda. Such structure of the DNA replication initiation complex may determine its lesser dependence on the processes occurring in the host cell, including transcriptional activation of the origin. Differences between molecular processes occurring during formation of replication complexes in lambda and Stx phages may indicate the specialization of the latter phages and their adaptation to specific environmental conditions where quick genetic switches are crucial.

## Introduction

*Escherichia coli* constitutes crucial component of the natural microbiota of the human and warm-blooded animals intestine (Actis, 2014). Although the vast majority of *E. coli* strains are commensals, some of them can also be pathogenic to humans (Leimbach et al., 2013). Shiga toxin-producing *E. coli* (STEC) strains, synthesizing one of the strongest toxins, are among the most dangerous pathogens (Paletta et al., 2020). Infections with these strains are particularly dangerous, because they can cause severe complications, while available antibacterial therapies are often not effective against those pathogens (Karmali, 2018). The latter problem is caused by two factors: firstly, STEC strains are often resistant to many antibiotics, secondly, the use of antimicrobials may be counterproductive, as some of them may enhance expression of Shiga toxins genes. Thus, even if a STEC strain is susceptible to a particular therapeutic its application may cause production of sufficiently high level of toxins to evoke hemolytic uremic syndrome in patients, resulting in significant morbidity and mortality (Kakoullis et al., 2019; Mir and Kudva, 2019). There are examples of many outbreaks caused by STEC which resulted in severe complications in thousands of patients and relatively many deaths (Devleesschauwer et al., 2019; Kintz et al., 2019).

Shiga toxins, the main virulence factors of STEC, are encoded by the genes (called *stx* genes) present in genomes of Shiga toxin-converting bacteriophages (or Stx bacteriophages) rather than in bacterial chromosome (Łoś et al., 2011). Stx phages occur as prophages in STEC genomes, and *stx* genes, like vast majority of prophage genes, are silent in the lysogenic state (Krüger and Lucchesi, 2015). Under various stress conditions, transition of the phage from lysogenic into lytic development is possible which involves the excision of its genome and replication of phage DNA, followed by progeny virion production and cell lysis (Łoś et al., 2011; Licznerska et al., 2016). Simultaneously, extensive production of Shiga toxin occurs, while inhibition of phage DNA replication impairs expression of *stx* genes (coding for the toxins) (Nejman-Faleńczyk et al., 2012; Nowicki et al., 2013; Balasubramanian et al., 2019). Therefore, detailed knowledge on the regulation of DNA replication of Stx phages may contribute to better understanding of the mechanisms of pathogenicity of STEC strains.

Stx phages belong to the group of lambdoid bacteriophages which include the model virus of molecular biology – bacteriophage λ (Łoś et al., 2011). DNA replication of this phage has been well understood, but its regulation is still not clear (Węgrzyn et al., 2012). It was described as a complex process involving both phage elements and protein machinery of its bacterial host (Weigel and Seitz, 2006). The key role in the regulation of initiation of this process is played by the bacteriophage-encoded O protein and transcriptional activation of the *origin* (Taylor and Wegrzyn, 1995). λ DNA replication requires binding of the phage-encoded O protein to four DNA sequences in the *origin* of replication region, named iterons, and formation of the nucleoprotein structure called O-some. This is the crucial step in the initiation event, and provides the basis for formation of the replication complex (Taylor and Wegrzyn, 1995; Weigel and Seitz, 2006).

**FIGURE 1:**
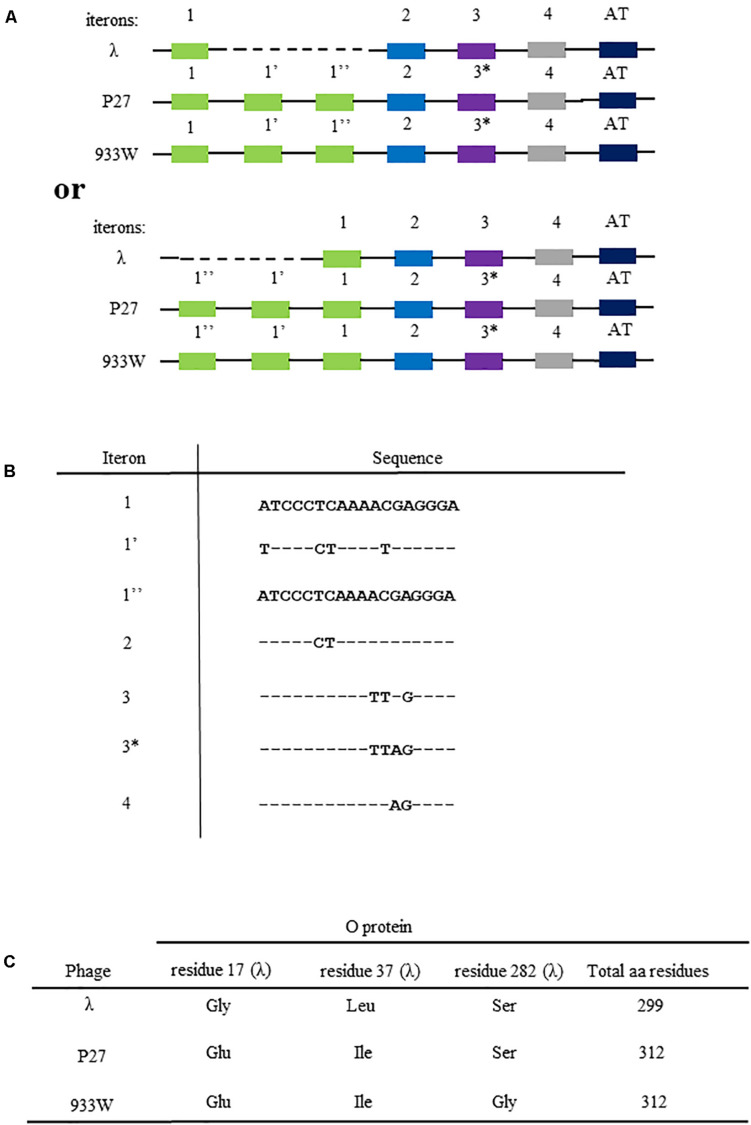
Differences in the nucleotide sequences of viral genome fragments that are involved in the initiation of DNA replication of bacteriophage λ and Stx phages (P27 and 933). Compositions of iterons and the AT-rich fragment are shown in **(A)** (since sequences of iterons 1 and 1″ are identical, two alternative ways of comparison between λ and Stx *origin* regions are possible, with dashed line indicating the missing fragment; the upper scheme presents the comparison as originally proposed by Nejman et al. (2009), and the lower scheme presents the alternative option). The scheme is not drawn to scale. Nucleotide sequences of iterons are shown in **(B)**, with (−) indicating bases identical to those occurring in iteron 1 and 1″, and letters indicating substitutions. Differences in amino acid sequences of O proteins of bacteriophage λ and Stx phages (P27 and 933W) are depicted in **(C)**.

It was demonstrated that the replication regions in genomes of Stx bacteriophages are similar to that of bacteriophage λ (Nejman et al., 2009). The key elements in this region are the *p*_R_ promoter, and *O* and *P* genes (coding for replication proteins) with the *origin* sequence (consisting of O-binding sequences, called iterons, and the AT-rich tract) located in the middle of the former gene (Figure 1). Only a few alterations were found between replication regions of λ and Stx phages, but they are responsible for significant differences in the DNA replication regulation. Particularly, various requirements for host-encoded factors, like DnaA and DksA proteins, and different dependence on the transcriptional activation of the *origin* (the process demonstrated previously as crucial for the control of the replication initiation; see Szalewska-Pałasz et al., 1994; Szambowska et al., 2011) were reported (Nejman et al., 2011). These differences result in various responses of intracellularly developing λ and Stx phages to environmental conditions, including starvation (Nejman et al., 2011).

Replication regions of Stx phages contain 6 iterons, contrary to 4 iterons present in λ, and a few amino acid alterations can be find in O and P proteins between these phages (Figure 1), which nevertheless cause significant differences in the phage DNA replication regulation (Nejman et al., 2011; Nowicki et al., 2015; Kozłowska et al., 2017). Recently, an improved method for overproduction and purification of O proteins of Stx phages has been reported which allowed to identify their specific binding to the *origin* regions (Kozłowska et al., 2017). Nevertheless, details of molecular interactions between these O proteins and iterons remained unknown, especially in the light of the above described differences between λ and Stx phages. Therefore, in this work, we aimed to determine details of formation of complexes between O proteins and *origin* regions of Stx phages and compare them to those of λ.

## Materials and Methods

### Proteins

Overproduction and purification of O proteins derived from phages λ, P27 and 933W used in this study was described previously (Kozłowska et al., 2017).

### Bacterial Strains and Source Plasmids

*E. coli* DH5α (F^–^ Φ80*lacZ*ΔM15 Δ(*lacZYA-argF*) U169 *recA1 endA1 hsdR17*(r^–^, m^+^) *phoA supE44 thi-1 gyrA96 relA1* λ^–^) strain (Taylor et al., 1993; Chen et al., 2018), and plasmids pCB104cmr (λ phage-derived), p27cmr (P27 phage-derived) (Nejman et al., 2009), pET24a_wtO_histag, pET24a_P27O_histag, pET24a_933WO_histag, pUC18_oriλ, and pUC18_oriStx (Kozłowska et al., 2017), and pUC18 (Yanisch-Perron et al., 1985) were used for production of proteins and DNAs for *in vitro* experiments. *E. coli* MG1655 and its *dnaA46* and Δ*seqA* derivatives (Nejman et al., 2009) were used for transformation efficiency analysis, performed as described previously (Nejman et al., 2009).

### Construction of DNA Templates and Plasmids

O protein binding to single iterons was tested by EMSA assay, employing DNA fragments obtained by single stranded oligonucleotides hybridization. Respective primer sequences are presented in Table 1 (oligonucleotides 8–19). Single-stranded DNA fragments (synthetic oligonucleotides) for hybridization were ordered from Sigma Aldrich. Oligonucleotides were used at a final concentration 1 μg/ml. Hybridization buffer was prepared in a volume of 1 ml. Synthetic oligonucleotide hybridization buffer consisted of 0.1 ml of 1 M Na_3_PO_4_, 0.03 ml of 5 M NaCl and 0.002 ml of 0.5 M EDTA at pH 8. The hybridization was carried out in a thermocycler starting from temperature 98°C for 2 min, then 2 min at 80°C, another 2 min at 65°C and slow cooling down to 20°C. Following hybridization, double stranded DNA fragments were ligated with plasmid pUC18, previously linearized with *Bam*HI and *Hin*dIII (Thermo Scientific^TM^), and used for transformation of *E. coli* DH5α competent cells. Obtained plasmids were isolated from bacterial cells using plasmid isolation kit (Sigma Aldrich) and sequenced to confirm their proper construction. DNA fragments for EMSA or footprinting tests were obtained by PCR technique with use of specific primers (see Table 1 for details).

**TABLE 1:** Oligonucleotides.

No.	Oligonucleotide name	Sequence 5′–> 3′	Assay	PCR template
1.	Cy5_ori_EMSA_ATrich_for	[Cyanine 5]TCAAGCAGCAAGGCGGCATGTTTG	EMSA, forward	pUC18_oriλ and pUC18_oriStx
2.	ori_EMSA_ATrich_for	CACTTGCTGCCGCTCTGAATTGC	EMSA, reverse	
3.	ATrich_57GC_rev	GGATTCGCCAGAATTCTCGGACGAAGCAT CGCCTCAGCTCGCTTCGGTACTAGTGTCC GATGTGTCCC	EMSA, reverse with AT-rich region replaced with region of 57% GC content	
4.	Cy5_pUC18_iterony_for	[Cyanine 5]GGGCTGGCTTAACTATGCGGCATC	PCR primer, forward	
5.	pUC18_iterony_rev	GTTAGCTCACTCATTAGGCACCC	PCR primer, reverse	
6.	pUC18_liniowy_footprint_for	CTTCCGGCTGGCTGGTTTATTG	Linearization of plasmid for footprinting	
7.	pUC18_liniowy_footprint_rev	CTTTATCCGCCTCCATCCAGTC	Linearization of plasmid for footprinting	
8.	Iteron1_pUC18_for	GATCCCGTAGCAGTCGCTGACATCCCT CAAAACGAGGGACGTAGCAGT CGCTGACA	Hybridization, forward	Not applicable
9.	it1_rev	AGCTTGTCAGCGACTGCTACGTCCCTC GTTTTGAGGGATGTCAGCGAC TGCTACGG	Hybridization, reverse	
10.	Iteron1×_pUC18_for	GATCCCGTAGCAGTCGCTGAGTTCCC CTAAAATGAGGGACGTAGCAGT CGCTGACA	Hybridization, forward	
11.	it1′_rev	AGCTTGTCAGCGACTGCTACGTCCCTC ATTTTAGGGGAACTCAGCGAC TGCTACGG	Hybridization, reverse	
12.	Iteron2_pUC18_for	GATCCCGTAGCAGTCGCTGAAATCCCCT AAAACGAGGGACGTAGCAGT CGCTGACA	Hybridization, forward	
13.	it2_rev	AGCTTGTCAGCGACTGCTACGTCCCTCG TTTTAGGGGATTTCAGCGAC TGCTACGG	Hybridization, reverse	
14.	Iteron3_pUC18_for	GATCCCGTAGCAGTCGCTGACATCCCTC AAATTGGGGGACGTAGCAGT CGCTGACA	Hybridization, forward	
15.	it3_rev	AGCTTGTCAGCGACTGCTACGTCCCCC AATTTGAGGGATGTCAGCGAC TGCTACGG	Hybridization, reverse	
16.	Iteron3^∗^_pUC18_for	GATCCCGTAGCAGTCGCTGACATCCCT CAAATTAGGGGACGTAGCAGT CGCTGACA	Hybridization, forward	
17.	it3^∗^_rev	AGCTTGTCAGCGACTGCTACGTCCCC TAATTTGAGGGATGTCAGCGAC TGCTACGG	Hybridization, reverse	
18.	Iteron4_pUC18_for	GATCCCGTAGCAGTCGCTGAATCCCTC AAAACAGGGGGACGTAGCAGT CGCTGACA	Hybridization, forward	
19.	it4_rev	AGCTTGTCAGCGACTGCTACGTCCCC CTGTTTTGAGGGATTCAGCGAC TGCTACGG	Hybridization, reverse	
20.	EMSA_for	[Cyanine 5]TGCTGCAAGGCGA	EMSA with single iteron sequence, forward	pUC18 with single iterons sequences (depending on iteron)
21.	EMSA_rev	CACTTTATGCTTCCGGCTCGTAT	EMSA with single iteron sequence, reverse	
22.	Footrinting for	AAGCAGCAAGGCGGCATGTTTG	Footprinting	pUC18_oriλ and pUC18_oriStx
23.	Footrinting rev	GCTGGTCAGAGGATTCGC	Footprinting	
24.	Fret	[Cyanine 5]CTGTTCTATTGTGATCTCTTATTAG [Cyanine 3]	FRET	Not applicable
25.	Fret_Donor_Cy3	CTGTTCTATTGTGATCTCTTATTAG[Cyanine 3]	FRET	
26.	FRET_methylation	CT(mp)G(mp) T(mp)TC(mp) T(mp)A(mp)T T(mp)G(mp)T(mp) GA(mp)T CTC TTA TTA G	EMSA with methylated ssDNA	
27.	Mut1Fd	GGACCAAATAAAAACATCTCAGAATGGTGC ATTCCTCAAAACGAAGGAGGTTCCCCTAAAATG	Site-directed mutagenesis of iteron sequence 1 (AT**C**CCTCAAAAACGA **G**GGAAT**T**CCTCAAAACGA**A**GGA)	p27R6K
28.	Mut1Rev	GATGTCCCTCATTTTAGGGGAACCTCCTTC GTTTTGAGGAATGCACCATTCTGAGATG	Site-directed mutagenesis of iteron sequence 1 (AT**C**CCTCAAAA ACGA**G**GGAAT**T**CCTCAAAACGA**A**GGA)	p27R6K
29.	Mut1 + 1′Fd	CATCTCAGAATGGTGCATTCCTCAAAACG AAGGAGGTTCTCCTAAAATGAGTGACATC CCTCAAAACGAGGG	Site-directed mutagenesis of iteron sequences 1 and 1′ (AT**C**CCT CAAAAACGA**G**GGAAT**T**CCTCAAAA CGA**A**GGA and TTC**C**CCTAAA ATGAG**G**GATTC**T**CCTAAAATGAG**T**GA)	p27mut1
30.	Mut1 + 1′Rev	GATGTTTTATCCCTCGTTTTAGGGGATTTT CCCTCGTTTTGAGGGATGTCACTCATTTT AGGAGAACCTCCT	Site-directed mutagenesis of iteron sequences 1 and 1′ (AT**C**CCTCA AAAACGA**G**GGAAT**T**CCTCAAAACGA **A**GGA and TTC**C**CCTAAAATGAG **G**GATTC**T**CCTAAAATGAG**T**GA respectively)	p27mut1
31.	Mut1′Fd	CATCTCAGAATGGTGCATCCCTCAAAACG AGGGAGGTTCTCCTAAAATGAGTGACATC CCTCAAAACGAGGG	Site-directed mutagenesis of iteron sequence 1′ (TTC**C**CCTAAAATGAG **G**GATTC**T**CCTAAAATGAG**T**GA)	p27R6K
32.	Mut1′Rev	GATGTTTTATCCCTCGTTTTAGGGGATTTTC CCTCGTTTTGAGGGATGTCACTCATTTTAGGA GAACCTCCC	Site-directed mutagenesis of iteron sequence 1′ (TTC**C**CCTAAAATGAG **G**GATTC**T**CCTAAAATGAG**T**GA)	p27R6K
33.	p27_IT1_Fd	[Cyanine5]GTGCATCCCTCAAAACGAGGGAGGTT	EMSA with wt and mutant variants in iteron 1	
34.	p27_IT1_Rev	AACCTCCCTCGTTTTGAGGGATGCAC	EMSA with wt variant in iteron 1 of P27	
35.	p27_IT1_prim_Fd	[Cyanine5]GAGGTTCCCCTAAAATGAGGGACATC	EMSA with wt variant in iteron 1′ of P27	
36.	p27_IT1_prim_Rev	[Cyanine5]GTGCATTCCTCAAAACGAAGGAGGTT	EMSA with wt variant in iteron 1′ of P27	
37.	p27_IT1mut_Rev	AACCTCCTTCGTTTTGAGGAATGCAC	EMSA with the mutant variant in iteron 1 of P27	
38.	p27_IT1mut_Rev	AACCTCCTTCGTTTTGAGGAATGCAC	EMSA with the mutant variant in iteron 1 of P27	
39.	p27_IT1_prim_mut_Fd	[Cyanine5]GAGGTTCTCCTAAAATGAGTGACATC	EMSA with the mutant variant in iteron 1′ of P27	
40.	p27IT1_prim_mut_Rev	GATGTCACTCATTTTAGGAGAACCTC	EMSA with the mutant variant in iteron 1′ of P27	

Derivatives of the p27cmr plasmid, bearing additional *origin* of replication of the R6K plasmid, were constructed as follows. The PCR fragment bearing *origin* R6K was obtained after the reaction with pCAH56 plasmid and primers *OriR6KFd* (5′ – GCC GAG CTC CAT CCC TGG CTT GTT GTC C) and *OriR6KFd* (5′ – GAT GAG CTC GAT CCG GCC ACG ATG CGT C). The reaction product was cut with *Sac*I, and ligated with the *Sac*I fragment of the p27cmr plasmid. Changes in iteron sequences 1 and/or 1′ of p27R6K plasmids, which did not alter the O amino acid sequence, were introduced by site-directed mutagenesis, producing p27mut1, p27mut1′, and p27mut1 + 1′ plasmids, respectively, using suitable primers (Table 1, primers 27–32). *E. coli* BW25142 (*lacI*^q^ *rrnB3 lacZ4787 hsdR* D(*araBAD*) D(*rhaBAD*) *phoBR580 rph-1 galU95 endA9 uid*A:*pir-116 recA1*) strain was used for plasmid replication after site-directed mutagenesis.

### Electrophoretic Mobility Shift Assay (EMSA)

All electrophoretic mobility shift assays (EMSA) were performed in the same manner. Indicated amounts of purified O proteins were incubated with 100 ng of different DNA fragments (1 μg in case of ssDNA fragment – oligonucleotides 26 in Table 1) in a freshly prepared O incubation buffer (0.2 M sodium phosphate pH 7.4, 0.1 M NaCl, 0.1 mM EDTA, 10 mM MgCl_2_, 1 mM DTT, 5% glycerol) containing 2 μg of poly dI:dC (final volume of the reaction mixture was 20 μl). Incubation was performed for 15 min at 30°C, and it was stopped by placing samples on ice, and addition 1 μl of 40% saccharose. 1% agarose gel electrophoresis was run in 0.5 × TBE at 4°C, 80 V, for 3.5 h. Gels were visualized with the use of Typhoon FLA 7000 scanner (GE Healthcare).

### Dimethyl Sulfide (DMS) Footprinting

#### Labeling of Oligonucleotides With ^32^P Isotope

Labeling of oligonucleotides for the primer extension reaction with a ^32^P phosphorus isotope was performed using polynucleotide kinase (PNK) (Thermo Scientific). Reagents (oligonucleotides, Buffer A, γ^32^P-ATP, T4 PNK – in amounts added according to the PNK manufacturer’s instruction) were mixed and incubated for 25 min at 37°C. Then, 10 μl of 50 mM EDTA were added to the mixture and incubation was continued for 10 min at 75°C. Following incubation, the solution was applied to Sephadex G-50 (Sigma Aldirch) resin. The labeled oligonucleotides were purified on the resin by spinning for 5 min at 800 rpm. The efficiency of the labeling reaction was assessed by measuring the radiation of 1 μl of purified oligonucleotide sample with a Geiger counter (minimum 1000/μl).

#### Obtaining DNA Template for Primer Extension Reaction

In order to prepare a modified plasmid DNA template for primer extension reactions, 1 μg of plasmid DNA (pCB104cmr or P27cmr) were incubated with proteins λ O, P27 O and 933W O for 15 min at 30°C (to achieve different molar ratios: 1:2, 1:4, 1:8, 1:16, and 1:32) in the incubation buffer [see section “Electrophoretic Mobility Shift Assay (EMSA)”]. Following addition of 1 mM dimethyl sulfide (DMS) to the mixture, the incubation was continued for 5 min at 30°C. Reaction was halted by addition of 100 μl ice-cold stopping buffer (3 M ammonium acetate, 1 M β-mercaptoethanol, 20 mM EDTA) and 300 μl of 96% ethanol. DNA samples were then incubated at −80°C for 30 min. Samples were centrifuged for 30 min at 15,000 rpm at 4°C. The pellets were washed gently with 300 μl of 70% ethanol and centrifuged again under above described conditions. Precipitates were dried using a Vacufuge Concentrator (Eppendorf). Pellets were suspended in 100 μl of 1 M piperidine solution and incubated for 30 min at 90°C to break methylated DNA bonds. 100 μl of the solution were applied to Sephacryl S-500 resin (Sigma Aldirch), and centrifuged for 5 min at 500 rpm.

#### DNA Primer Extension Reaction

Primer extension reaction with Taq polymerase (Thermo Fisher Scientific) was carried out using an isotope-labeled oligonucleotide and modified plasmid DNA template under the conditions specified by Taq polymerase manufacturer. Following incubation scheme was applied: 3 min at 92°C, then 40 cycles of denaturation for 30 s at 92°C, annealing for 50 s at 50°C, and extension for 1 min at 72°C, and then final extension for 2 min at 72°C.

#### Sanger Sequencing Reaction

The sequencing reaction was carried out using the DNA Cycle Sequencing Kit (Jena Bioscience), according to the manufacturer’s instructions.

#### DNA Electrophoresis

The 8% polyacrylamide gel was prepared using 50 ml of TBE buffer, 100 ml 40% polyacrylamide 19:1 mixture, 240 g urea and 150 ml ultrapure water. The mixture was slightly heated to dissolve urea and degassed using a vacuum pump. To 80 ml of the mixture 640 μl of 10% APS and 30 μl of TEMED were added, and the mixture was poured between glass plates of the electrophoresis apparatus. The gel polymerization lasted 1 h at room temperature. DNA sample after the primer extension reaction and sequencing was dried in a heating block at 99°C for 20–30 min. Obtained pellet was suspended in 12 μl of Loading Buffer (95% formamide, 20 mM EDTA, 0.05% bromophenol blue, 0.05% xylene cyanol FF) and 5 μl of the solution were applied onto the polyacrylamide gel. Electrophoresis was carried out in a previously heated (45–50°C) gel, at 80 W, 1900 V, and 60 mA for 3.0–3.5 h. Following electrophoresis, gel was rinsed for 5 min in water, then 5 min in 10% acetic acid and again for 5 min in water. The gel was then dried, and sealed with an ionizing radiation capture screen (GE Healthcare). The exhibition was carried out overnight. Screen signal was visualized in the Typhoon FLA 7000 scanner (GE Healthcare).

### FRET Assay

15 pM of ssDNA fragment with donor (Cy3) and acceptor (Cy5) was incubated with 500 ng of purified O proteins in Incubation buffer (0.2 M sodium phosphate pH 7.4, 0.1 M NaCl, 10 mM MgCl_2_, 1 mM DTT, 0.1 mM EDTA, 5% glycerol) in a final volume of 20 μl for 5 min at room temperature. Excitation was conducted at 530 nm (Cy3) and fluorescence (550–700 nm wavelength spectrum) was detected with the use of EnSpire^TM^ Multimode Plate Reader (Perkin Elmer).

## Results

The *origin* of replication of lambdoid bacteriophages, located in the middle of the *O* gene, consists of sequences which bind the O replication initiator protein, called iterons, and the region containing large proportion of AT base pairs (the AT-rich region). As indicated in Figure 1A, the *origin* of bacteriophage λ DNA replication, *ori*λ, contains 4 iterons, while *origins* of the Stx phages (exemplified in this work by phages P27 and 933W) contain 6 iterons. They are numbered as 1, 1′, 1″, 2, 3, and 4, where iterons 1, 2, 3, and 4 are present in λ, and iterons 1′ and 1″ occur only in Stx phages. There are a few nucleotide alterations between cognate iterons (Figure 1B). The sequence alignment is presented in Supplementary Figure S1. All iterons are almost the same in all tested *origin* regions (when compared between phages), except one nucleotide difference in iteron 3 of Stx phages relative to that of λ. Thus, this Stx-specific iteron has been named 3^∗^. However, since iterons 1 and 1″ have identical sequences, there are two possible ways of comparing the replication regions of λ and Stx phages (Figure 1A). The presence of 2 additional iterons in DNA of Stx phages results in appearance of 13 additional amino acid residues in the O protein, relative to phage λ *O* gene product (Figure 1C). Moreover, the sequence of the O protein of phage P27 contains two alterations relative to λ O protein (Gly17Glu and Leu37Ile), and that of phage 933W contains three such alterations (Gly17Glu, Leu37Ile, and Ser282Gly) (Figure 1C). The O protein sequence alignment with secondary structure assignment is shown in Supplementary Figure S2. Moreover, potential secondary DNA structures formed at the iteron region are presented in Supplementary Figure S3. Considering significant differences in the regulation of DNA replication between λ and Stx phages (Nejman et al., 2009, 2011; Nowicki et al., 2015), we aimed to investigate the effects of the above mentioned alterations on formation of the nucleoprotein complexes by the O proteins of lambdoid phages and their *origin* regions.

**FIGURE 2:**
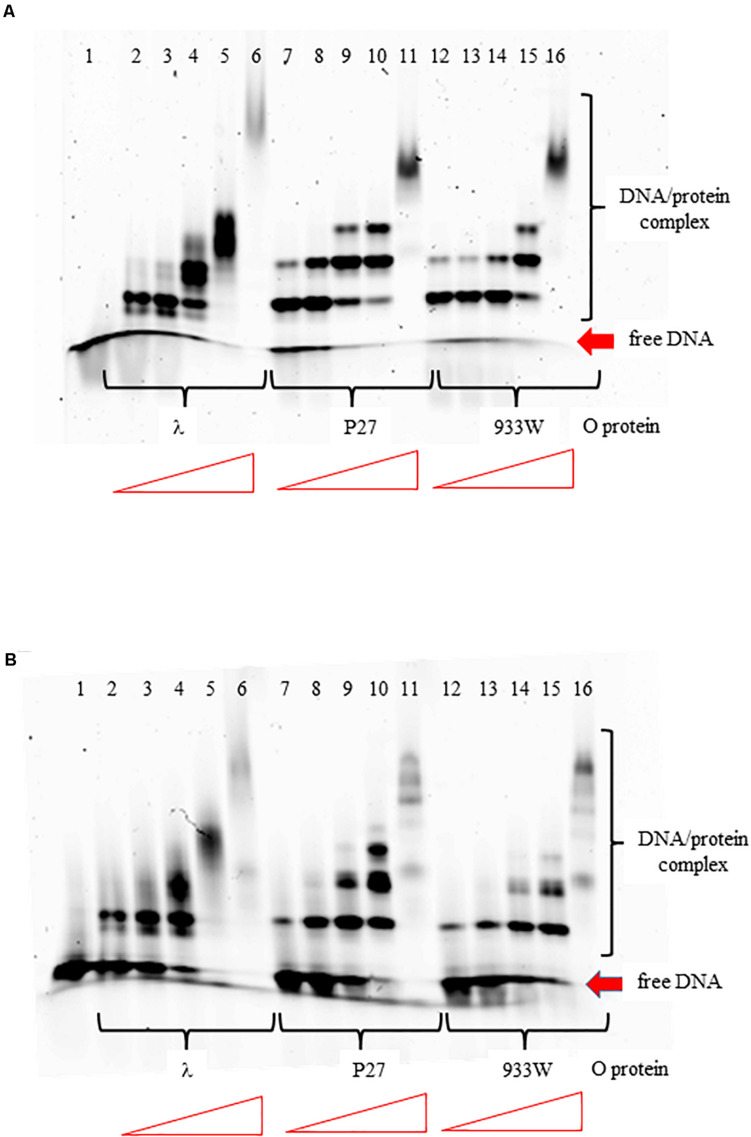
Interactions of O proteins of phages λ, P27 and 933W with DNA templates containing four panel **(A)** or six panel **(B)** iterons, as assessed by EMSA. Increasing molar ratios of DNA:O proteins, indicated in particular wells, were as follows: 1:2, 1:4, 1:8, 1:16, and 1:32. Lane no. 1 contains no O protein.

The O proteins of phages λ, P27, and 933W were purified as described previously (Kozłowska et al., 2017). Using EMSA assay with DNA fragments containing the replication *origin* regions of phages λ and P27 (with 4 and 6 iterons, respectively), we found that all tested O proteins formed multiple complexes with both templates (Figure 2). However, binding efficiency to *origin* regions varied among the O proteins variants. It was also different for the two Stx O proteins dependent on whether the origin fragment containing 4 or 6 iterons was present in the binding reactions, whereas λO seemed to bind similarly to both origins. For the four iteron λ *ori* both Stx O proteins formed nucleoprotein complexes of lower mass slightly more efficiently, at lower protein concentrations, than the λO protein. However, as the protein concentration was increased, λO formed DNA-protein complexes of higher mass more readily than both the P27 and 933W O proteins. On the other hand, the two latter O proteins formed consecutive nucleoprotein complexes with their cognate origin with lower efficiency than the λO protein. The difference in binding efficiency in comparison to λO was higher for the 933 O protein than for its counterpart from the phage P27 (Figure 2). These results strongly suggest that amino acid alterations between various O proteins result in differences in efficiency of formation of their complexes with the *origin* region. However, it was difficult to verify in the EMSA test if all iterons in the Stx *ori* are bound by the O proteins at higher protein concentrations, since nucleoprotein complexes of high mass often formed diffused bands, migrating at variable positions, reflecting their lower stability, various conformations or protein aggregation. Discrete bands representing 4, 5, or 6 dimers of the O proteins bound could not be distinguished.

**FIGURE 3:**
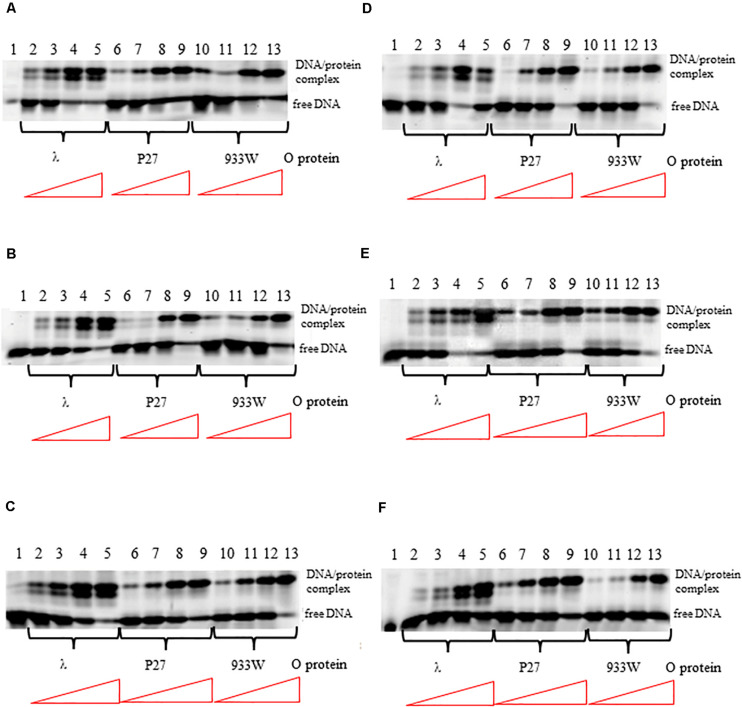
Interactions of O proteins of phages λ, P27 and 933W with DNA templates containing single iterons: iteron 1/1″ **(A)**, iteron 1′ **(B)**, iteron 2 **(C)**, iteron 3 **(D)**, iteron 3* **(E)**, and iteron 4 **(F)**. Increasing molar ratios of DNA:O proteins, indicated in particular wells, were as follows: 1:1, 1:2, 1:4, and 1:8. Lane no. 1 contains no O protein. Note that lanes 10/11 in panel A, and lanes 4/5 in panel D, are swapped.

The differences in interactions of O proteins derived from λ and Stx phages to *origin* regions might result from either various binding cooperativity properties of the initiator proteins or lower affinity of O proteins of Stx phages to iterons. Therefore, we tested efficiency of binding of O proteins to individual iterons, using templates containing single iterons: 1/1″, 1′, 2, 3, 3^∗^, 4. We found that all tested O proteins formed complexes with each tested iteron, however, efficiency of their formation were slightly different. Phage λ O protein forms two different complexes with each iteron while O proteins of Stx phages form predominantly one complex (Figure 3). Again, binding of the λ O protein was the strongest among tested proteins. Moreover, iterons 2 and 3^∗^ were bound with the highest efficiency by all tested O proteins, while formation of the nucleoprotein complexes was the weakest for iterons 1′ and 4 (Figure 3). Nevertheless, the differences between efficiency of binding of O proteins by individual iterons were not dramatic.

**FIGURE 4:**
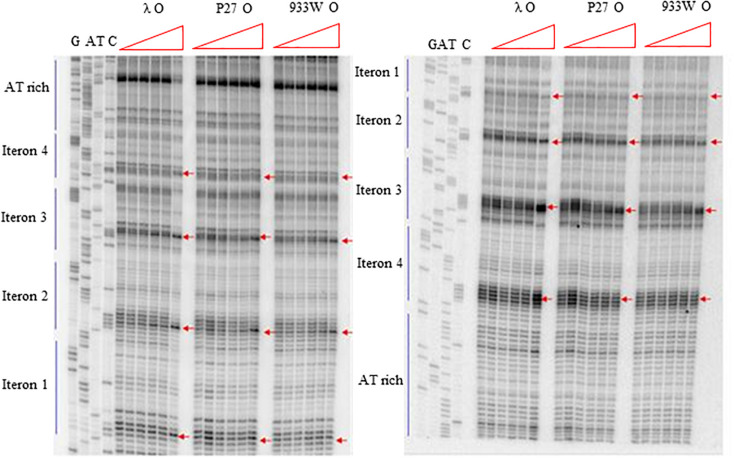
Interactions of O proteins of phages λ, P27 and 933W with DNA templates containing four iterons, as assessed by DMS footprinting. Increasing molar ratios of DNA:O proteins, indicated in particular wells, were as follows: 1:0 (no O protein), 1:2, 1:4, 1:8, 1:16, and 1:32. Arrows indicate major changes (enhancement) in bands’ intensities.

**FIGURE 5:**
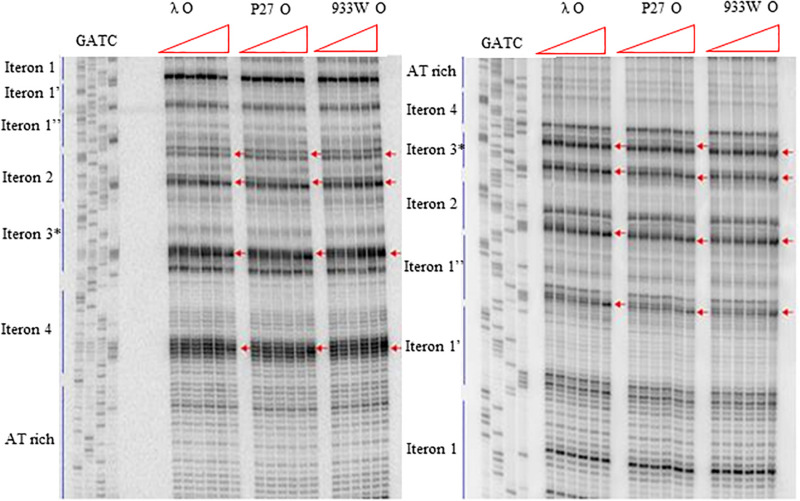
Interactions of O proteins of phages λ, P27 and 933W with DNA templates containing six iterons, as assessed by DMS footprinting. Increasing molar ratios of DNA:O proteins, indicated in particular wells, were as follows: 1:0 (no O protein), 1:2, 1:4, 1:8, 1:16, and 1:32. Arrows indicate major changes (enhancement) in bands’ intensities.

More detailed information about binding of the O proteins to iterons were obtained in footprinting experiments. Results of experiments with both types of *origin* regions (with 4 and 6 iterons) confirmed the efficiency of binding of different variants of the O protein, with λ O being the most effective and the 933W O protein the least effective in interactions with iterons (Figures 4, 5 for results of experiments with the templates containing 4 and 6 iterons, respectively). In experiments with the template containing four iterons, all O proteins bound iterons 2 and 3 with the highest efficiency, then iteron 1, and iteron 4 with the lowest efficiency (Figure 4). Protection of the DNA sequence, indicating the contact sites with the O protein, is evident on both sides of the template, and includes two distal G residues in the G-tracks present in each iteron. Enhancement of the signal, indicating DNA bending, can be seen also at both template sides, between O-bound residues and in the middle of each iteron (Figure 4). These results are in agreement with previously published observations (Zahn and Blattner, 1985). Importantly, when the template containing six iterons was used, only four of them were bound efficiently by O proteins in each experimental system (i.e., with the O protein of phages λ, P27 and 933W). Sequences of two proximal iterons were not clearly protected by any of the tested O proteins, though weak interactions of iteron 1′ with O at the highest protein concentrations could be observed (Figure 5). Therefore, we conclude that the arrangement of iterons in the Stx phage *origin* relative to λ should be represented as indicated in the lower part of Figure 1A. At higher protein concentrations, λO protein bound iteron 1′, although with lower efficiency than the four iterons proximal to the AT-rich region, and very weakly interacted with the most distant iteron 1″. On the other hand the P27 and W933 O proteins showed even weaker footprint on iteron 1′, with very low level of protection and moderate enhancement at the correct nucleotides. The signal of binding was very weak at protein concentrations which resulted in full occupancy of the consecutive iterons 1/1″, 2, 3, and 4 by both proteins (Supplementary Figure S4). Similar, but even weaker pattern was observed with respect to all O proteins at the 1/1″ iteron situated at the edge of the origin.

Iterons are intrinsically bent sequences (Zahn and Blattner, 1985) and their anisotropy could affect binding parameters of the O protein. To assess whether curvature could underlie slight differences in binding of the O proteins to individual iterons, we performed an analysis of iterons geometry using AA Wedge model (Goodsell and Dickerson, 1994). The data obtained with DNA Curvature Analysis online application^1^ showed that iterons 1/1″, 1′, 2, and 4 have identical local bending pattern. On the other hand, iterons 3 and 3^∗^, which contain a track of three A, instead of four present in the remaining iterons, have slightly different curvature from the rest but indistinguishable from each other (Supplementary Figure S5). This suggests that differences in intrinsic DNA bending between individual iterons do not account for the observed preferences of the O proteins to particular sequences present in the origin context.

Using the tool mentioned above, we also analyzed DNA curvature of the entire *origin* regions, both containing 4 and 6 iterons. The results of the analysis (Supplementary Figures S6, S7) indicate that both types of origins contain a significantly curved region encompassing iterons 1–3. Both iteron 4 and especially iterons 1′–1″ are separated by regions of low curvature from iterons 1–3. The DNA structure of phage origins could be related to the observed hierarchy of iterons occupation by the O replication initiators, with iterons 4 and 1′–1″ being the least preferred.

**FIGURE 6:**
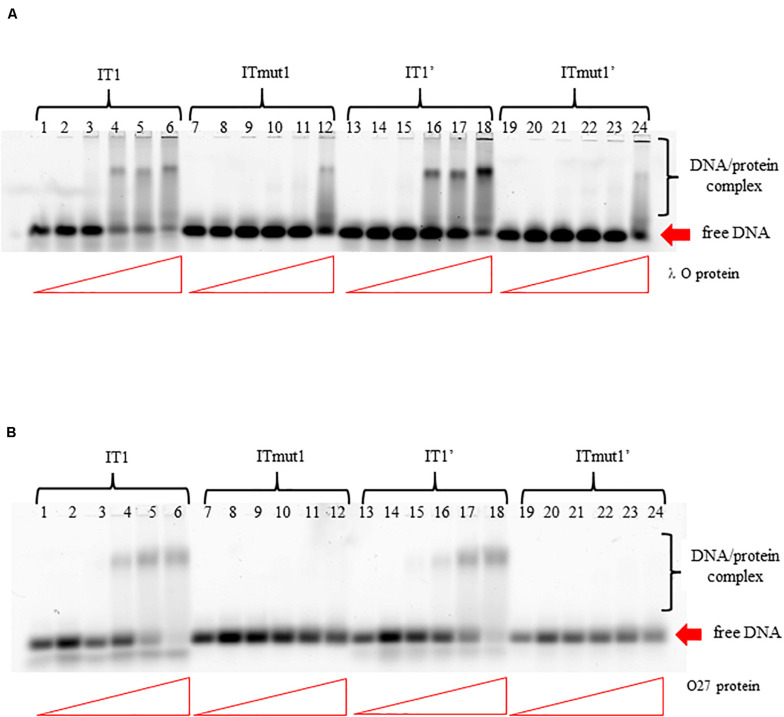
Interactions of O proteins of phages λ panel **(A)** and P27 panel **(B)** with DNA templates containing various versions of iterons (IT), as assessed by EMSA. Templates with wild-type iterons 1 or 1′ (IT1 or IT1′, respectively) and with mutant iterons 1 or 1′, unable to bind O proteins (ITmut1 or ITmut1′, respectively) were used. Increasing molar ratios of DNA:O proteins, indicated in particular wells, were as follows: 1:2, 1:4, 1:8, 1:16, and 1:32. Lanes no. 1, 7, 13, and 19 contain no O protein.

To test if the additional iterons occurring in the *origin* regions of Stx phages are functionally significant, we have constructed plasmids bearing disrupted iteron 1 and/or 1′ sequence(s). DNA templates bearing such mutations bound λ O only very weakly at the highest used amounts of the protein, and did not bind the P27 O protein at all (Figure 6). Some differences in results of experiments with wild-type iterons may be due to differences in the DNA fragment lengths (55 vs. 28 bps). The presence of two bands of free DNA for wild-type iterons but only one band for mutated ones is apparently because of formation of bending in wild-type sequence which disappears when the mutation is present. To test functionality of the iteron 1′, double-*origin* plasmids have been constructed which contained lambdoid (P27-derived) replication region and *origin* of plasmid R6K, active only in the presence of the active *pir* gene. Sequences of iterons 1 and 1′ were changed as described above, and the alterations of base pairs were chosen to obtain disrupted O-binding properties and unchanged amino acid sequences of the O proteins, encoded by the *O* genes (as mentioned, the *origin* is located in the middle of the *O* gene). Replication of plasmids containing either wild-type or mutated iterons was possible in the *E. coli* strain expressing the *pir* gene due to activity of the *ori* R6K, irrespective of the activity of lambdoid *origin*. However, transformation of *E. coli* cells devoid of the *pir* gene was possible only in the presence of the active lambdoid *origin.* We have tested efficiency of transformation by lambdoid plasmids bearing either wild-type iterons or those with mutations in iteron 1 and/or 1′ of *E. coli* wild-type host, as well as *dnaA46*, Δ*seqA* or double *dnaA46* Δ*seqA* mutants. These mutants were used since it was previously demonstrated that: (i) incompatibility between λ plasmids and *dnaA* mutations arises from both inefficient stimulation of transcription from the *p*_R_ promoter, responsible for transcriptional activation of the *origin*, by the mutant DnaA protein and the competition between the lambda P protein and the host DnaA and DnaC proteins for DnaB helicase, (ii) both mechanisms must be operative for the incompatibility, and (iii) the incompatibility is abolished in the absence of the *seqA* gene function (Glinkowska et al., 2001) as the *seqA* gene product modulates *p*_R_ activity (Słomińska et al., 2003a) and interplays with DnaA at this promoter (Słomińska et al., 2003b). We found that disruption of either interon 1 or iteron 1′ or both abolished ability of lambdoid plasmid to transform *E. coli* wild-type strain. The presence of mutation(s) in *dnaA, seqA* or both these genes did not change the plasmid inability to transform host cells (Table 2). We conclude, that both iteron 1 and iteron 1′ are necessary for efficient replication of lambdoid plasmids derived from Stx phages, despite only weak interactions of the latter iteron with the O protein.

**TABLE 2:** Transformation efficiency of E. coli wild-type, dnaA, seqA, and dnaA seqA hosts by derivatives of p27cmr plasmids bearing mutated iteron sequences 1 and 1′ at 30°C. Results are presented as mean values of three independent experiments ± SD.

Plasmid	Efficiency of transformation (transformants/μg of plasmid DNA)			
	dnaA*^+^*seqA*^+^*	dnaA46 seqA*^+^*	dnaA^+^ ΔseqA	dnaA46 ΔseqA
p27R6K	5.38 ± 1.37 × 10^4^	3.79 ± 0.39 × 10^4^	<10^1^	7.00 ± 3.49 × 10^2^
p27mut1	<10^1^	<10^1^	<10^1^	<10^1^
p27mut1′	<10^1^	<10^1^	<10^1^	<10^1^
p27mut1 + 1′	<10^1^	<10^1^	<10^1^	<10^1^

**FIGURE 7:**
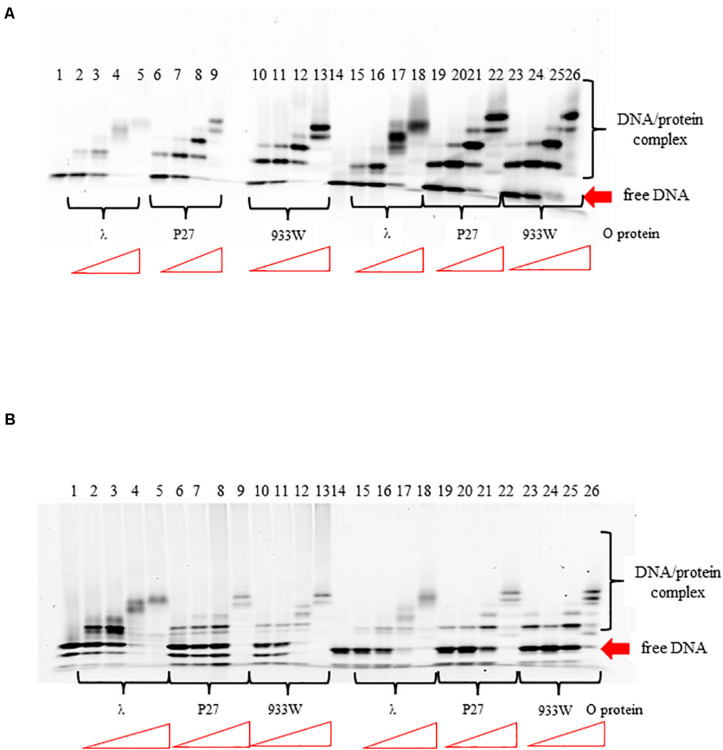
Interactions of O proteins of phages λ, P27 and 933W with DNA templates containing four panel **(A)** or six panel **(B)** iterons together with the AT-rich region (lanes 1–13) or the AT-rich region replaced with a sequence contained 57% of GC base pairs (lanes 14–26), as assessed by EMSA. Increasing molar ratios of DNA:O proteins, indicated in particular wells, were as follows: 1:2, 1:4, 1:8, and 1:16. Lanes no. 1 and 14 contain no O protein.

In the lambdoid phage *origin* of replication, iterons are flanked by the AT-rich region (see Figure 1A). To test if the AT-rich sequence influence binding of the O protein to iterons, we have replaced the original AT-rich region with that containing 57% GC base pairs. We found that such a change did not influence efficiency of binding of O protein to iterons (Figure 7). Patterns of formed nucleoprotein complexes were also not influenced by replacement of the AT-rich region with a neutral sequence. This was true for both templates, containing either four or six iterons (Figures 7A,B, respectively). The presence of two bands for free DNA (Figure 7B, lines 1–14) and the presence of six protein-DNA bands (line 26) can be explained by formation of secondary structured by ssDNA fragments.

**FIGURE 8:**
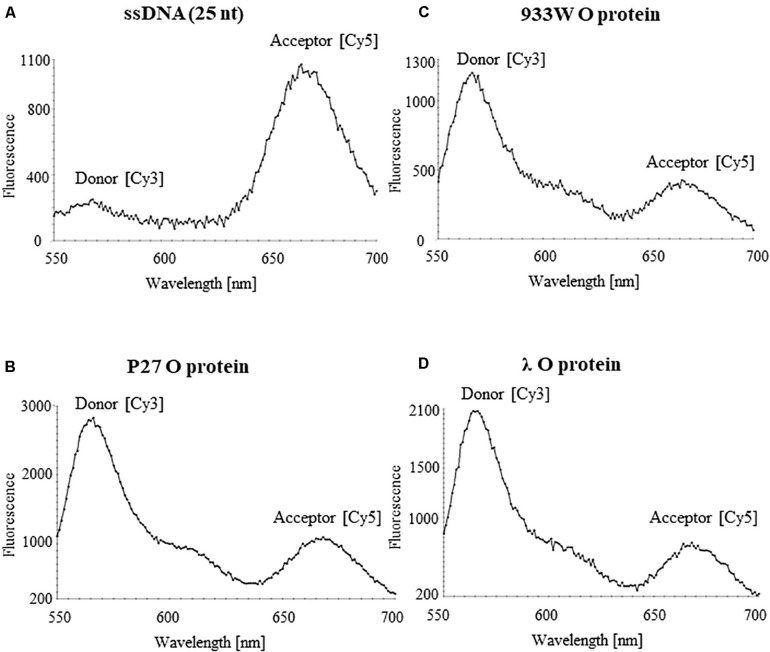
Interactions of O proteins of phages P27 **(B)**, 933W **(C)**, and λ **(D)**, with ssDNA templates containing the AT-rich region as assessed by FRET. Results obtained with the sample containing only the ssDNA template are shown in **(A)**.

Binding of phage λ O protein to single stranded DNA (ssDNA) has been demonstrated previously (Learn et al., 1997). Therefore, we have asked if all tested O proteins can interact with ssDNA of the AT-rich region. The analysis was performed using FRET technique. ssDNA was labeled with Cy3 and Cy5 fluorescent dyes forming the donor-acceptor pair. For the free ssDNA, donor excitation resulted in effective energy transfer and an increase in acceptor fluorescence emission concomitant with a decrease of the donor fluorescence. Binding of the O proteins caused reduction of FRET efficiency observed as an increase of the donor signal and diminution of the acceptor fluorescence. Results of these experiments indicated that O proteins of phages λ, P27 and 933W can efficiently interact with ssDNA, indeed (Figure 8). The fluorescence signals were similar in all experiments in which O proteins (from λ, P27 and 933W) were present (Figure 8), suggesting that the investigated mechanism does not differ between tested phages.

## Discussion

Since DNA replication of Stx phages appears to be necessary for effective production of Shiga toxins by STEC strains (Nejman-Faleńczyk et al., 2012; Nowicki et al., 2013; Balasubramanian et al., 2019), understanding of the regulation of this process is crucial not only for basic science but also for applications related to development of novel anti-STEC treatments. Although replication regions of genomes of Stx phages are highly similar to that of bacteriophage λ, there are important differences (summarized in Figure 1 and Supplementary Figures S1–S3) which result in significantly altered regulation of Stx phage DNA replication relative to λ (Nejman et al., 2009, 2011; Nowicki et al., 2015). Therefore, we decided to investigate details of molecular interactions between the initiator O proteins of Stx phages with the *origin* regions, using purified proteins and nucleic acids.

Previously published results (Zahn and Blattner, 1985, 1987; Schnos et al., 1988) indicated that the 19 bp (imperfect) inverted repeat of the iteron is the minimal sequence required for efficient binding by the λ O protein. At low protein concentrations, only the inner two iterons were bound, while binding to all four iterons occurred at higher protein concentrations. The O protein binds to the replication origin in both linear and circular (relaxed) forms, but *origin* unwinding requires the negatively supercoiled DNA form introduced by the host gyrase. Results of our experiments indicated that O proteins of Stx bacteriophages bind specifically to iterons in the *origin* regions (Figure 2; see also Kozłowska et al., 2017), however, these interactions are weaker relative to binding of bacteriophage λ O protein to its specific DNA target sequences (Figure 2). Apparently, amino acid alterations in O proteins of Stx phages relative to the λ O protein, listed in Figure 1C and indicated in Supplementary Figure S2), are responsible for these differences in their affinity to iterons. Although each individual iteron can be bound by any of tested O proteins (encoded by λ, P27 or 933W phages) (Figure 3), when the whole *origin* region was considered, only four iterons were involved in effective interactions with O protein molecules, irrespective of the presence of either four or six such DNA sequences in the template (Figures 4, 5). Nevertheless, two additional iterons (1′ and 1″) in *origin* of Stx phage can be bound by the O protein and are necessary for effective DNA replication (Figure 6, Table 2, and Supplementary Figure S4).

The AT-rich region appears to be dispensable for formation of the O-some structure (a specific nucleoprotein structure containing the O protein and iterons) (Figure 7). In fact, it was demonstrated (Dodson et al., 1986) that two iterons and the adjacent AT-rich region form the minimal DNA fragment competent for unwinding. Moreover, for *in vitro* replication of *ori*λ-based plasmids, only the two iterons proximal to the AT-rich region were required (Wickner and McKenney, 1987). Since this AT-rich tract is crucial for DNA unwinding during replication initiation, and all tested O protein variants revealed similar ability to interact with single stranded DNA rich in A and T residues (Figure 8), one may suggest that the mechanism of DNA stretching is the same for all tested phages.

The presence of additional two iterons in *origin* sequences of Stx phages relative to λ results also in appearance of additional 13 amino acid residues in corresponding O proteins, as iterons are located in the middle of the *O* gene (Figure 1). One might ask if such additional protein fragment can influence interaction between O and its DNA target. The secondary structure prediction of O proteins (Supplementary Figure S2) suggests that the additional protein fragments are not involved in interactions with DNA. Regarding the role of these two additional iterons in Stx phages, on one hand, it was reported that three iterons are sufficient for efficient replication of phage λ, as suggested by the normal growth and burst size of the mutant phage (Moore et al., 1981). On the other hand, additional iterons were found here to be necessary for transformation of P27-derived plasmids (Table 2). Therefore, one might propose that iterons 1′ and 1″ ensure either elevation of the local concentration of the O protein or stabilization of the O-some which can be crucial due to lower affinity of Stx O proteins to iterons, relative to λ O. Such a model is compatible with the already described mechanism of DNA-protein interactions which usually requires a proximity of A residue stretch in DNA (Rohs et al., 2009), and such a motif is actually present in iterons located in the *origin* of lambdoid phage replication (see Figure 1B).

There is also a question about potential roles of hairpin and cruciform structures, potentially formed by the replication region of lambdoid phages. Roles of such structures in DNA replication have been discussed previously (Bikard et al., 2010). In fact, each iteron in lamboid *origin* is an inverted repeat, and in the sequences of the entire gene O, the biggest and most stable hairpin structures are encoded by the iterons 2 and 4, 1′ and 4, and 2 and 3 (Supplementary Figure S3). It appears that duplication of the iterons in Stx pahges may influence folding of the potential hairpin/cruciform structures. This may influence efficiency of binding of the O protein and formation of the O-some, and in fact, Stx replication regions are bound less efficiently than that of λ. Therefore changes in secondary DNA structures might be an additional role of additional iterons in Stx phages.

Recent studies indicated that DNA replication may significantly influence the major developmental decision of bacteriophage λ, whether to produce progeny phages and lyse the host cell or to lysogenize it (Shao et al., 2018). Since such decision is crucial for all lambdoid phages, one can speculate that λ and Stx phages evolved in somewhat different ways to produce optimal regulatory systems functioning in environments they occur in. Less stringent binding of the O protein to iterons, and the presence of additional iterons which can modulate formation of the O-some, possibly in response to various environmental conditions, might be profitable for Stx phages which have to quickly change the developmental pathway. Gastrointestinal tract of ruminants is their primary habitat in which STEC strains developed a specific Stx phage-dependent strategy to fight unicellular eukaryotic predators, like ciliates (Łoś et al., 2013). Therefore, ability to adjust the genetic switch between lysogenic and lytic developmental options rapidly may require more flexible regulation of DNA replication initiation, ensured by modulatory effects of additional iterons and weaker binding of the O protein to them. On the other hand, phage λ that was discovered as a parasite of the *E. coli* K12 strain (Lederberg and Lederberg, 1953), a bacterium originally isolated from human stool and known to occur in human and animal intestine as well as in natural waters (Kuhnert et al., 1995; Na et al., 2006). Hence, this phage might prefer more strict developmental decisions which can be effectively made when employing stronger interactions between the O initiator protein and the replication *origin*. This strategy may ensure more unequivocal lysis-vs.-lysogenization decision which is compatible with the proposal of Shao et al. (2018) about the coupling of phage DNA replication to the λ developmental choice.

## Data Availability Statement

The raw data supporting the conclusions of this article will be made available by the authors, without undue reservation, to any qualified researcher.

## Author Contributions

KK participated in planning experiments, performed most of them, participated in data interpretation, and drafting the manuscript. MG participated in planning experiments, data analysis, and revising the manuscript. LB participated in constructing of plasmids, performing EMSA, conducting experiments with mutant plasmids, performing DMS footprinting experiments, and revising the manuscript. LG participated in data analysis, preparation of figures, and revising the manuscript. JD assisted in EMSA experiments. GW was principal investigator of the project, supervised the work, and drafted the manuscript. All authors contributed to the article and approved the submitted version.

## Conflict of Interest

The authors declare that the research was conducted in the absence of any commercial or financial relationships that could be construed as a potential conflict of interest.

## Footnotes

1 https://www.lfd.uci.edu/∼gohlke/dnacurve/
