# Supplementary figures and images for: Formation of Complexes Between O Proteins and Replication Origin Regions of Shiga Toxin-Converting Bacteriophages
Source: Front Mol Biosci. 2020 Aug 19;7:207. doi: 10.3389/fmolb.2020.00207 (PMC7466680; doi:10.3389/fmolb.2020.00207)

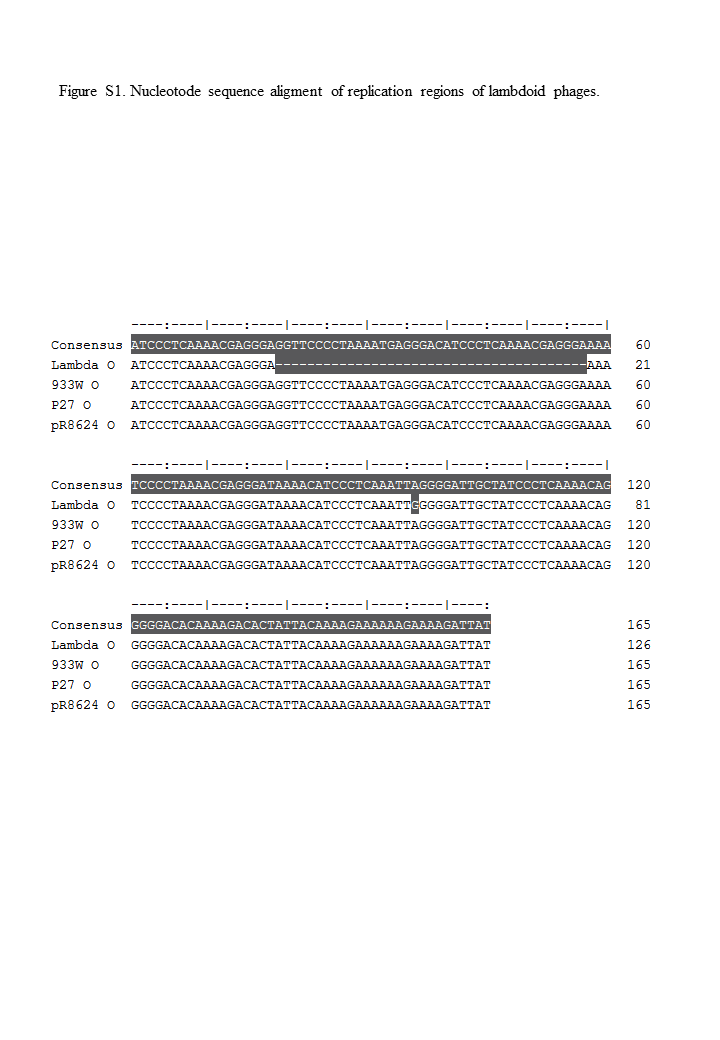

Supplement: Supplementary file 1 [file Image_1.TIF]

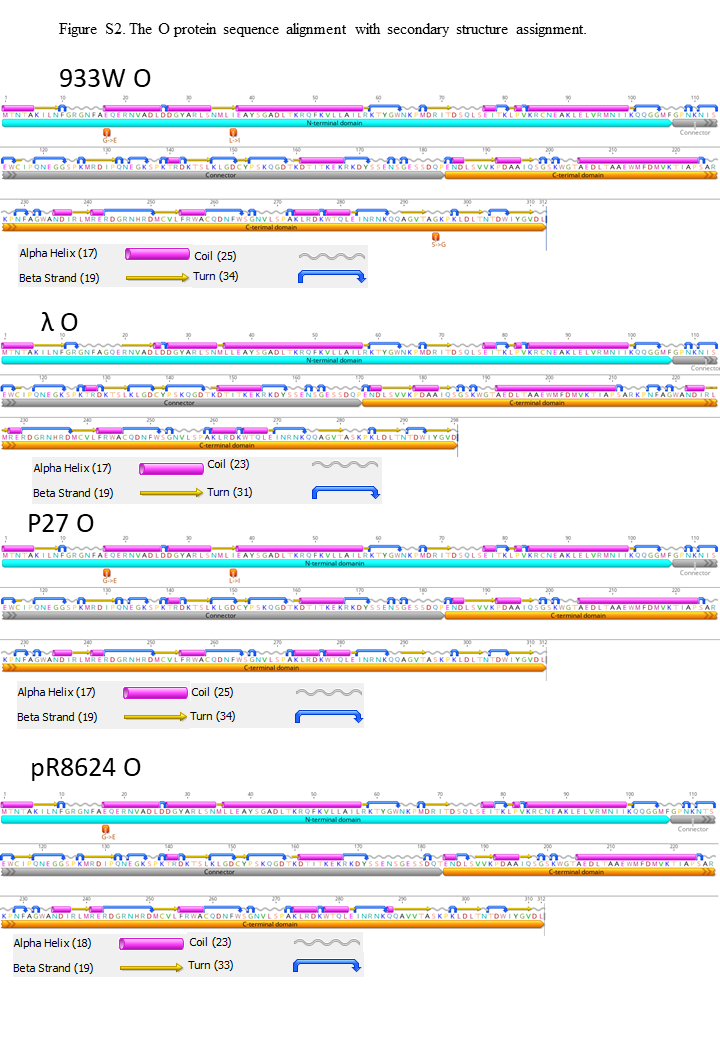

Supplement: Supplementary file 2 [file Image_2.TIF]

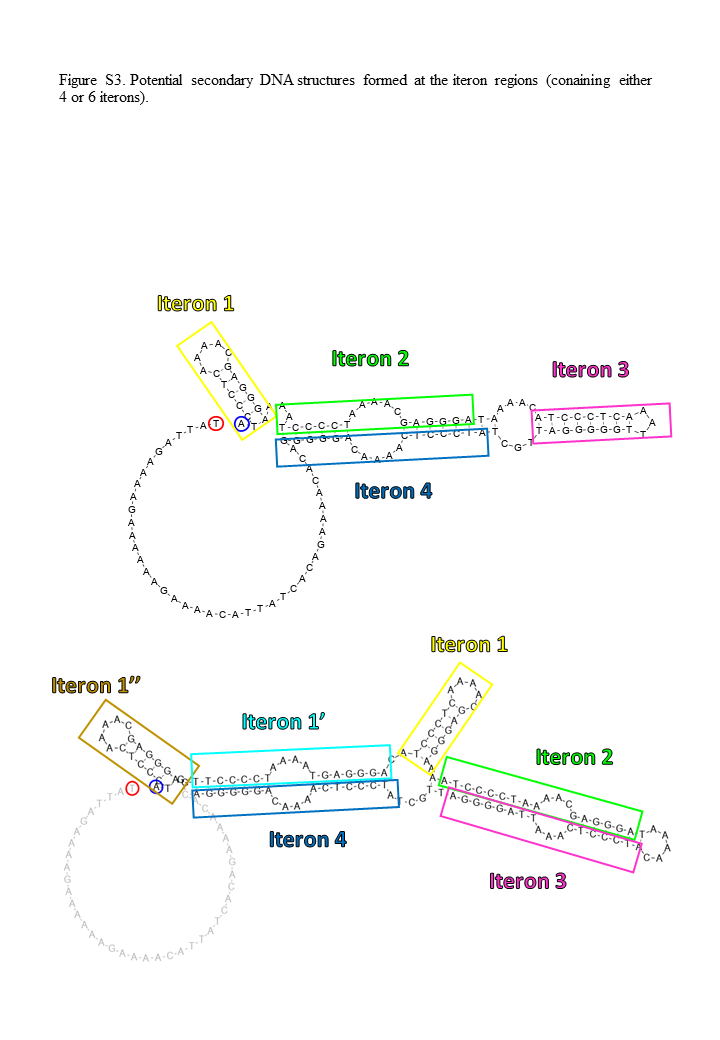

Supplement: Supplementary file 3 [file Image_3.TIF]

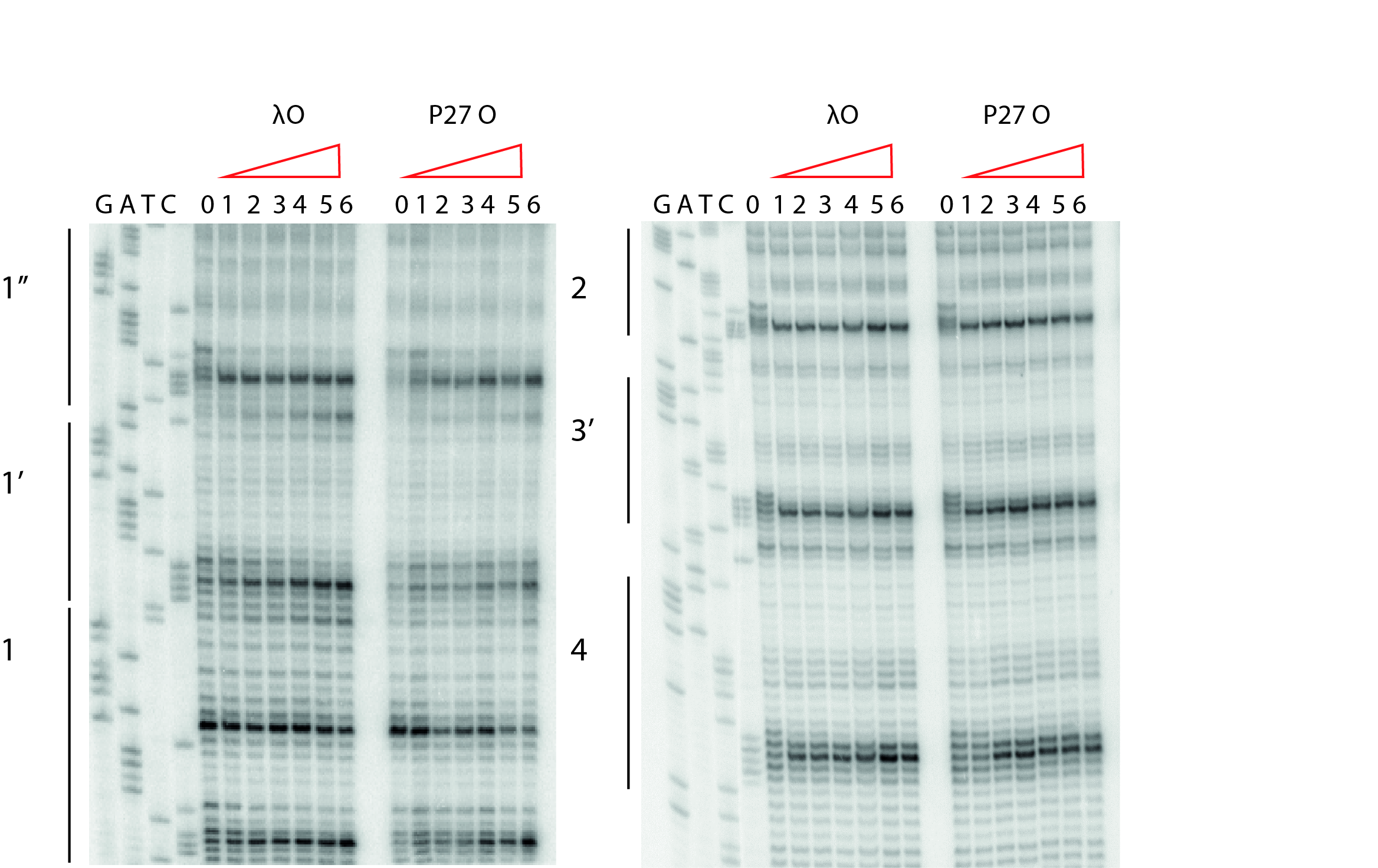

Supplement: Supplementary file 4 [file Image_4.TIF]

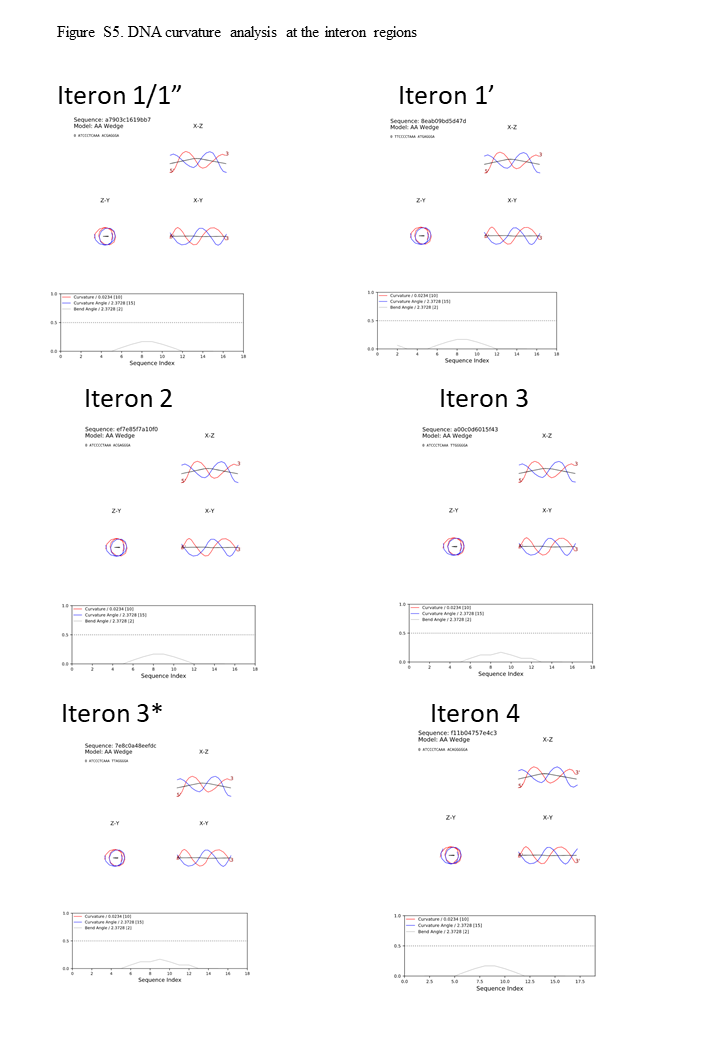

Supplement: Supplementary file 5 [file Image_5.TIF]

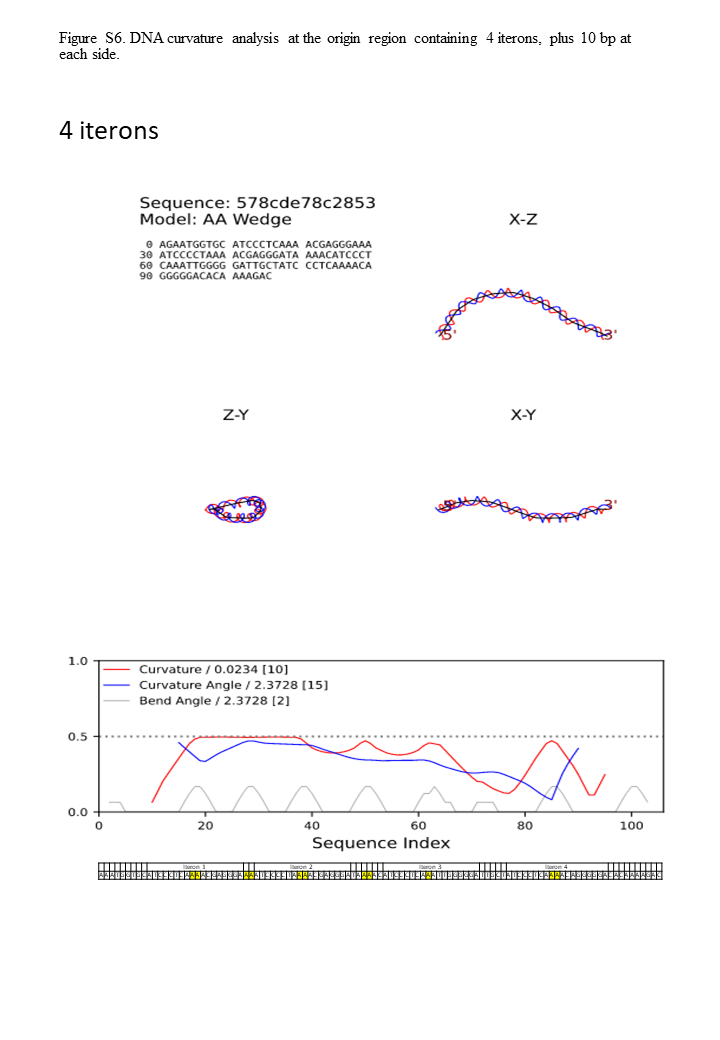

Supplement: Supplementary file 6 [file Image_6.TIF]

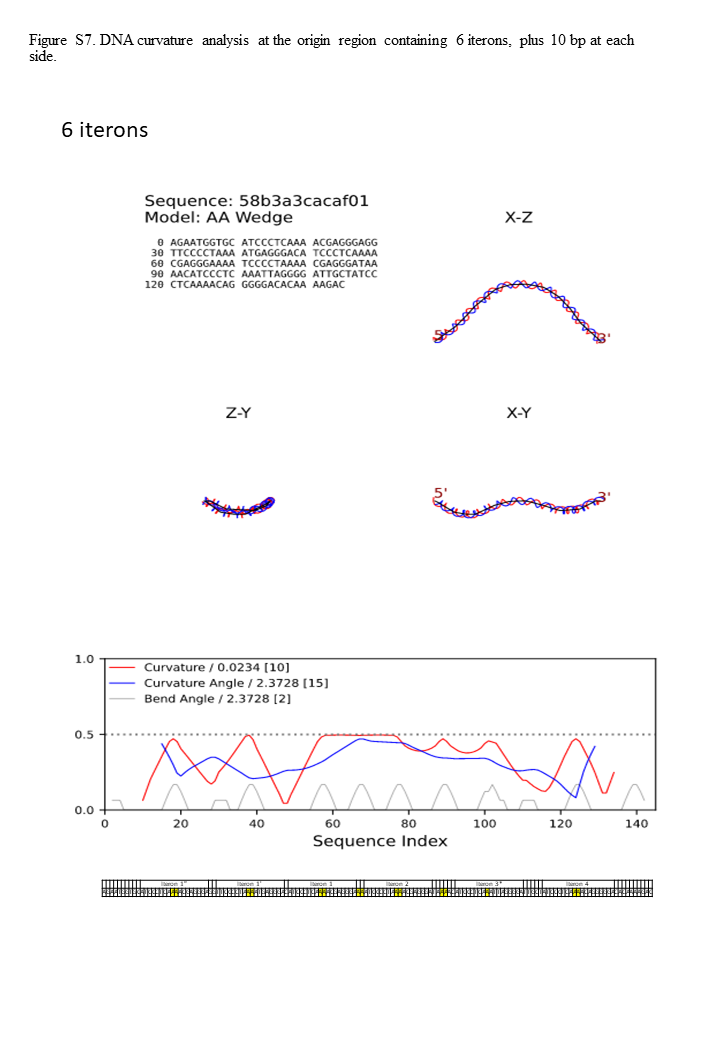

Supplement: Supplementary file 7 [file Image_7.TIF]
