# Supplementary material for: Formation of Complexes Between O Proteins and Replication Origin Regions of Shiga Toxin-Converting Bacteriophages
Source: Front Mol Biosci. 2020 Aug 19;7:207. doi: 10.3389/fmolb.2020.00207 (PMC7466680; doi:10.3389/fmolb.2020.00207)
Supplement: Supplementary file 8 [file Data_Sheet_1.pdf]

**Legends to Supplementary Figures**

**Figure S1.** Nucleotide sequence alignment of replication regions of lambdoid phages.

**Figure S2.** The O protein sequence alignment with secondary structure assignment.

**Figure S3.** Potential secondary DNA structures formed at the iteron regions (containing either 4 or 6 iterons).

**Figure S4.** Interactions of O proteins of phages λ and P27 with DNA templates containing six iterons, as assessed by DMS footprinting. Increasing amounts of O proteins (0, 10, 20, 30, 40, 60, 80 pmol) were present in consecutive reactions (wells 0-6). Position of each iteron along the DNA strand was indicated by vertical bars.

**Figure S5.** DNA curvature analysis at the iteron regions.

**Figure S6.** DNA curvature analysis at the *origin* region containing 4 iterons, plus 10 pb at each side.

**Figure S7.** DNA curvature analysis at the *origin* region containing 6 iterons, plus 10 pb at each side
